# Universal Approach to FRAP Analysis of Arbitrary Bleaching Patterns

**DOI:** 10.1038/srep11655

**Published:** 2015-06-25

**Authors:** Daniel Blumenthal, Leo Goldstien, Michael Edidin, Levi A. Gheber

**Affiliations:** 1 The Avram and Stella Goldstein-Goren Department of Biotechnology Engineering, Ben Gurion University of the Negev, Beer-Sheva, ISRAEL; 2 Department of Biology, Johns Hopkins University, Baltimore, MD, USA

## Abstract

The original approach to calculating diffusion coefficients of a fluorescent probe from Fluorescence Recovery After Photobleaching (FRAP) measurements assumes bleaching with a circular laser beam of a Gaussian intensity profile. This method was used without imaging the bleached cell. An empirical equation for calculating diffusion coefficients from a rectangular bleaching geometry, created in a confocal image, was later published, however a single method allowing the calculation of diffusion coefficients for arbitrary geometry does not exist. Our simulation approach allows computation of diffusion coefficients regardless of bleaching geometry used in the FRAP experiment. It accepts a multiple-frame TIFF file, representing the experiment as input, and simulates the (pure) diffusion of the fluorescent probes (2D random walk) starting with the first post-bleach frame of the actual data. It then fits the simulated data to the real data and extracts the diffusion coefficient. We validate our approach using a well characterized diffusing molecule (DiIC_18_) against well-established analytical procedures. We show that the algorithm is able to calculate the absolute value of diffusion coefficients for arbitrary bleaching geometries, including exaggeratedly large ones. It is provided freely as an ImageJ plugin, and should facilitate quantitative FRAP measurements for users equipped with standard fluorescence microscopy setups.

Fluorescence recovery after photobleaching (FRAP) is a classical biophysical method, that has been used extensively to study molecular diffusion, particularly in cell membranes. It consists of irreversibly photobleaching a fluorescently labeled diffusing species, and subsequently following the fluorescence recovery over time. Fluorescence recovery then can be used to characterize diffusion of bleached molecules out of, and unbleached molecules into the bleached area and so extract the diffusion coefficient (D) of the fluorescent species. Extensive reviews^1^,^2^,^3^,^4^, describe the wide range of applications FRAP has found during the years. While the experimental setup though specialized is relatively straightforward, the interpretation of the results is complex, severely limiting the quantitative use of the technique. Originally, FRAP assumed photobleaching by a well-characterized Gaussian profile laser beam and measurement of fluorescence recovery with a strongly attenuated version of the very same Gaussian beam. This approach offers well-defined and relatively convenient initial and boundary conditions, allowing a closed-form solution of the diffusion equation^5^. The method, however, requires a very precise characterization of the bleaching laser beam, a technically challenging task.

With the introduction of digital imaging and the Laser Scanning Confocal Microscope (LSCM), a multitude of imaging variations of the technique became possible. For example:

1. bleaching a circular region using a Gaussian laser beam, while monitoring the fluorescence recovery of the entire cell using wide field illumination and a fast CCD camera.
2. bleaching a rectangular area with a scanning (Gaussian) laser beam and monitoring the recovery with a scanning beam, that images an area much larger than the bleached area (typical LSCM FRAP experiment)
3. bleaching a (rectangular) shape as above with a scanning (Gaussian) beam and monitoring recovery of the whole field of view using a spinning disk confocal microscope (CCD camera)

Importantly, all these approaches can be implemented on commercially available microscopes, making the FRAP technique accessible to cell biologists as well as biophysicists. Indeed, commercially available confocal laser scanning microscopes (CLSM) have standard, push-button, FRAP abilities, thus a recovery curve can be easily extracted from the stack of images, following bleaching of any shape for any duration and with any intensity. In this realm, the shape of recovery curves from the very same sample can be very different, for different shapes of bleached area and different bleaching durations. These unknown, uncharacterized and uncontrolled initial and boundary conditions are often too complex to allow a precise closed, analytical solution of the diffusion problem^6^; a closed form solution does not exist even for a rectangular bleaching area, and an empirical one for calculating diffusion coefficients from a rectangular bleaching geometry, appropriate for imaging-FRAP in a confocal microscope, was later published^7^. This approximate solution allows quantitative FRAP for rectangular shapes of bleached areas. However, in commercially available equipment it is easily possible to choose initial conditions (e.g., size of bleached area, time of bleaching) which violate the assumptions required for even approximate solutions, and thus obtain calculated values of D that differ by orders of magnitude from the actual molecular diffusion coefficient. All these problems limit in fact the range of quantitative FRAP applications.

Alternative methods to FRAP have been developed, which do not have the problem of determining bleaching geometry. Light sheet microscopy based Fluorescence Correlation Spectroscopy (FCS) creates maps of protein diffusion coefficients across an entire cell^8^. An extension of the FCS method, Raster Image Correlation Spectroscopy (RICS)^9^,^10^,^11^, uses a CSLM to rapidly measure many focal points within a cell during a raster scan, and allows for accurate measurement of diffusion coefficients. Finite Element (FE) methods are also applied in conjunction with FRAP simulations to extract diffusion coefficients^12^.

While the alternatives to FRAP produce excellent results for a variety of geometries, they are highly specialized and some even require dedicated, homemade systems for implementation. We have developed an alternative approach to extracting diffusion coefficients directly from FRAP image stacks. Rather than approximate diffusional recovery from the data for a limited set of initial conditions, we run a 2D stochastic simulation of recovery and fit it to the observed data. The use of stochastic simulations in the assessment of diffusion has been previously used to theoretically investigate the source of anomalous diffusion of protein in the plasma membrane^13^ and the ability to detect this mode of diffusion in FRAP measurements^14^. We are using simulation in order to predict the recovery over time of an experimentally acquired bleached region, and then, comparing this prediction with the real (experimentally acquired) recovery, we extract the absolute diffusion coefficient that leads to best fit of the simulated recovery to the real one. To do this, we have developed a fast algorithm that allows the extraction of diffusion coefficients, given a stack of images representing a FRAP experiment. This approach was previously used by Wedekind *et al.* for a specialized mode of FRAP they termed SCAMP^15^. Our method may be regarded as a generalization of that approach, allowing the user to use FRAP data acquired with any method, for a completely arbitrary geometry of the initially bleached area.

## Results

### Simulation Approach for FRAP Analysis

**Figure 1:**
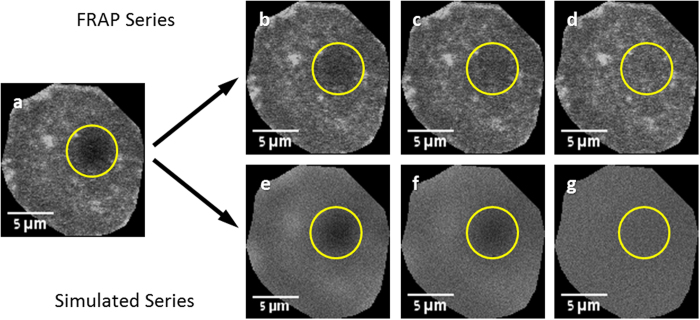
FRAP recovery series and its corresponding simulated recovery (**a**) the first post-bleach frame is used as the basis of the simulated recovery. (**b**–**d**) Images from the actual FRAP series showing recovery of the bleached spot. (**e**–**g**) Images from the simulated recovery series, showing simulation progression. The circle represents the user-chosen area for monitoring fluorescence recovery.

Our generalized algorithm, for any FRAP geometry, (freely available as an ImageJ plug-in http://imagej.nih.gov/ij/plugins/sim-frap/index.html) assumes *pure diffusion* in two dimensions (additional effects such as flow, binding reactions, etc. could be added in the future). The algorithm takes the first post-bleach frame for implicit initial and boundary conditions, and simulates two-dimensional random walk of each “molecule” in each pixel, starting with this time point (Fig. 1). Thus, no characterization of the bleaching profile is required, since this information is encoded in the image. In this approach, the lack of closed analytical solutions for complex geometries is not a limiting factor, since the diffusion equation is effectively solved numerically by iterative simulation. Diffusion coefficients are extracted from stacks of hundreds of frames and thousands of pixels per frame in a few minutes.

### Validation of the approach

To validate the simulation approach, we performed FRAP measurements on cell membranes stained with the lipid analog DiI. The DiI lipid analogs are a well-characterized family of fluorescent probes^16^,^17^,^18^. We chose specifically DiIC_18_(3) as its long hydrophobic alkyl chains allow for strong association with the plasma membrane thus once inserted, traffic as an integral part of it. DiIC_18_ has been used to measure diffusion in plasma membranes of a variety of cells^19^,^20^,^21^,^22^,^23^, and thus serves as a good comparison standard for quantitative measurement of diffusion coefficients in plasma membranes; it is also known to perform pure diffusion, thus conforms with our simulation assumption.

**Figure 2:**
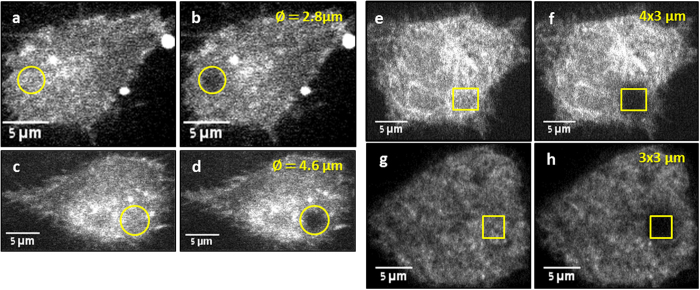
Different bleaching patterns of DiI stained CCL-1.3 cells Two examples of circular beam bleaching pattern (**a**–**d**), before bleaching (**a**,**c**) and after bleaching (**b**,**d**); beam diameter is specified in the top right corner. Two examples of rectangular bleaching patterns (**e**–**h**), before bleaching (**e**,**g**) and after bleaching (**f**,**h**); box size is specified in the top right corner.

We first bleached circular areas of various diameters and rectangular areas of various dimensions (Fig. 2), and compared the results obtained using the analytical equations available for these cases, with the results obtained from the simulation approach. Having established a good agreement, we proceeded to bleach arbitrary and awkward shapes, for which no analytic solution exists and compared with the previously obtained results, to show that the same diffusion coefficient is obtained (as expected).

### D from equation and D from simulation are similar, for circular and rectangular bleaching geometries, and independent of bleached area

#### Circular bleaching

**Figure 3:**
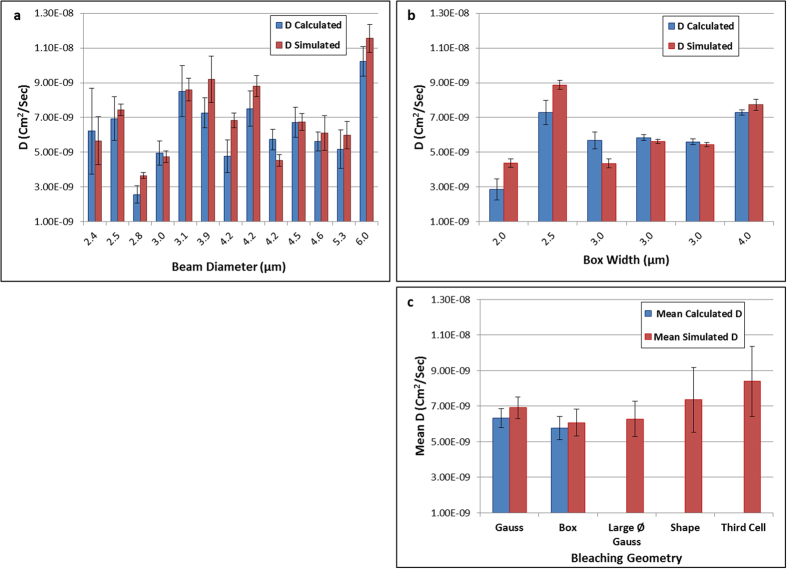
Comparison of Calculated and Simulation Extracted Diffusion Coefficient (**a**) Results of 13 single experiments using circular bleaching; the beam diameter is the value extracted from the post-bleach frame, using angular averaging. The resulting diffusion coefficient is clearly not correlated with the diameter of the bleached region. (**b**) Results of rectangular bleaching pattern. Box width (w) was left as a free fitting parameter in fitting Eq. (3) to the data. Error bars account for uncertainty in the determination of beam diameter (Gauss) or width (Box) (for analytical calculation) and iteration time (for simulation). (**c**, two leftmost columns) Comparison of mean (over all experiments: 13 for circular and 6 for rectangular) diffusion coefficient extracted with the analytical models, with the simulation approach. The mean differences between calculated (blue) and simulated (red) diffusion coefficients are statistically insignificant. (**c**, three rightmost columns) the mean diffusion coefficient of a large diameter Gaussian bleached spot, arbitrary shape and a third of a cell calculated using the simulation approach. Error bars represent SEM.

Thirteen different cells were bleached with a Gaussian beam with diameter of 2.4 *μm* < ∅ < 6.0 *μm*. Since our algorithm simulates simple diffusion, it is important to confirm that the measured DiI diffusion coefficient does not depend on the size of bleached area. Indeed, we observe no correlation between D and bleached area, as shown in supplementary figure S1. In Fig. 3a, we compare individual values for calculated - *D*_*c*_ (using equations (2) and (3), Supplementary material), and simulated - *D*_*s*_ (using equations (1), Supplementary material) diffusion coefficients. Error bars for the analytically calculated *D*_*c*_ account for uncertainty in the determination of beam diameter and for fitting error in extraction of the time constant, as further explained in the Supplementary material. Error bars for the simulation extracted *D*_*s*_ result mainly from the fitting of the simulated data to the real one, and thus determination of iteration time (see Supplementary material fur further elaboration). While analyzing the results presented in Fig. 3a, it is important to remember that “the diffusion coefficient” of a species in the realm of cells is usually determined only at the level of an order of magnitude, from a single experiment. More precise determination is normally impossible, due to the variability between cells and even between areas of the same cell. This well-known phenomenon is demonstrated also in our case, in Fig. 3a, for three different cells bleached with the same 4.2 μm circular spot. These three bars represent in fact a triplicate repetition of the same experiment, with precisely the same parameters, on three different cells, and the diffusion coefficient calculated according to the classical Axelrod method (blue columns). The differences between these three results are statistically significant, meaning that they originate in the sample itself (and not in the experimental accuracy, depicted by the error bars). This observation applies obviously to the rest of the measurements presented in Fig. 3, many of which are statistically different from each other. Despite this heterogeneity, averaging over many values, acquired on different cells and preferably with various parameters, results in more stable values of *D*, representative of a diffusing species. This can be observed in Fig. 3c where average values of *D* measured using either a circular, or a box bleaching geometry are compared side-by-side (the blue columns labeled “Gauss” and “Box”) and show insignificant differences.

In comparing our simulation approach with the results from analytical formulae (whether for circular or rectangular bleaching geometries), we note that, while *D*_*c*_ and *D*_*s*_ are in some cases significantly different for *single* experiments (Fig. 3a and Fig. 3b), it is obvious that a). neither *D*_*c*_ nor *D*_*s*_ depend on the dimension of the bleached area in a systematic way, thus confirming DiI is performing pure diffusion in the membrane and b). *D*_*s*_ is closely following *D*_*c*_ showing a good correlation. The cell-to-cell variation in *D* values within the 13 cells examined is larger than the differences between *D*_*c*_ and *D*_*s*_ for each single experiment. Therefore, in order to get a representative value for the diffusion coefficient of DiI in our cells, we present the averaged values in Fig. 3c. The mean diffusion coefficient for the Gaussian bleaching pattern, determined using the closed equation was 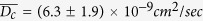, and that determined from our simulation, 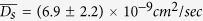 (Fig. 3c, leftmost columns labeled “Gauss”), with an insignificant statistical difference between them (two tailed t-test, p_gauss_ = 0.47).

#### Rectangular bleaching

Six different rectangular (box) bleaching series were analyzed (Fig. 3b), with dimensions between 2.5 × 2.5 μm^2^ to 4 × 4 μm^2^. Error bars for the analytical calculation (*D*_*c*_) account for uncertainty in the determination of box width and error bars for the simulation (*D*_*s*_) result mainly from the determination of iteration time (for further elaboration, see Supplementary material). As seen in Fig. 3b, *D*_*s*_ closely follows *D*_*c*_ and they correlate well. As before, a triplicate repetition on three different cells is presented, with an identical 3 μm × 3 μm box width. Here too, the results calculated according to the analytical formula are not statistically identical, albeit the cell-to-cell variation is smaller than in the circular bleaching experiments, mainly due to the larger bleached (and probed) area, which inherently averages over cell inhomogeneities. To compare between the analytical formula method and the simulation approach we turn our attention to the average values, presented in Fig. 3c and labeled “Box”. The mean calculated diffusion coefficient (using equation (4), Supplementary material) for the box bleaching experiments was 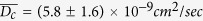 and the value determined from our simulation was 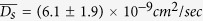, (Fig. 3c, columns labeled “Box”), with no significant statistical difference (two-tailed t-test, p_box_ = 0.32).

### D from simulation, for arbitrary bleaching geometry, is similar to D from calculation

**Figure 4:**
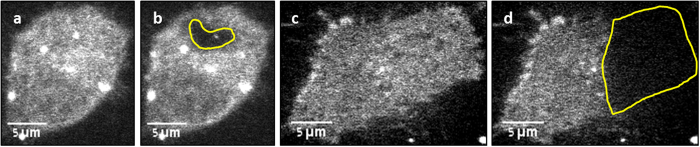
Examples of Arbitrary Shape Bleaching Patterns Before and after images of DiI stained CCL 1.3 cells (**a**,**b**) Bleaching a random shape pattern included within the cell. (**c**,**d**) Bleaching about a third of the cell.

Having established that the simulation approach yields statistically similar results compared with the traditional analytical methods for bleaching geometries, for which such methods exist, we further used our simulation approach to calculate the diffusion coefficient from bleaching an arbitrary shape (contained within the area of one cell), about a third of the area of a cell (both shown in Fig. 4), or an exaggeratedly large diameter circular area (shown in supplementary figure S2). The two last shapes wildly violate the assumptions on which any FRAP analytical model is based (the bleached number of molecules should represent a minute fraction of the total), and are “forbidden” by any best-practice guides on FRAP measurements. Attempting to use classical, closed-form solutions to extract diffusion coefficients of such large circular areas, results in errors as demonstrated in supplementary figure S2. There we bleached circular areas of increasing diameters, and extracted diffusion coefficients by naively applying the approach described in equations (2),(3) (Supplementary material). We show that bleaching more than ~25% of the total cell area yields discrepancies of up to one order of magnitude.

Three arbitrary shape experiments (one example is shown in Fig. 4a,b) were analyzed, resulting in a mean diffusion coefficient of 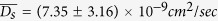 (shown in Fig. 3c, fourth column, labeled “Shape”). Three third cell area experiments were analyzed (one example is shown in Fig. 4c,d), resulting in a mean diffusion coefficient 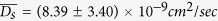 (shown in Fig. 3c, rightmost bar labeled “Third cell”). Three large diameter Gaussian bleaching experiments were analyzed (example shown in supplementary figure S2), resulting in a mean (simulation-extracted) diffusion coefficient of 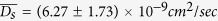 (shown in Fig. 3c, third column labeled “Large Ø Gauss”). All three results are statistically similar (one-way ANOVA, Tukey post-hoc, p = 0.61) to the ones calculated and extracted in the previous experiments (Fig. 3c, “Gauss” and “Box”).

### Stochastic fluctuation of diffusion coefficient extracted by simulation

The value of D as predicted by the simulation may vary between repeated simulations with the same image stack. The pseudo-random generator employed in programming languages, at the heart of diffusion simulation, may (and should) yield different results in different runs, thus a different “reality” may be simulated in different runs. We checked this aspect, by running the simulation on the same image stack for 10 different times. Seven different stacks, representing seven independent FRAP experiments were analyzed this way (10 runs on each one of the seven stacks), and the divergence of D values from their mean (as a result of different runs) was compared with the error of individual D values (calculated from fitting the simulated curve to the real data, see supplementary material). In all cases combined, the mean divergence resulting from different runs on the same data is of the order of ~2.5%, while the mean of the relative errors of individual D values (the uncertainty in D value, stemming from uncertainty in the fitting of simulated data to the experimental data) was ~7.5% (data not shown). The first is an expression of the stochastic character of the simulation (for each run, a slightly different recovery curve is obtained), while the latter is a measure of “unexplained variation” around the model, representing measurement noise and deviations of the real data from the ideal assumptions of the simulation. Thus, the uncertainty in determining D from a single experiment is larger than the statistical uncertainty owing to “chance”. Stochastic noise of the simulations itself does not significantly contribute to the total uncertainty of D calculated this way, and it is of no real use to run the algorithm more than once, to obtain robust and stable values of D, for one experiment. It is, however, important to calculate a representative D for the diffusing species in the membrane of the given cell, by averaging multiple values of D obtained from multiple, independent experiments performed on different cells.

### Sensitivity to selection of monitored area

**Figure 5:**
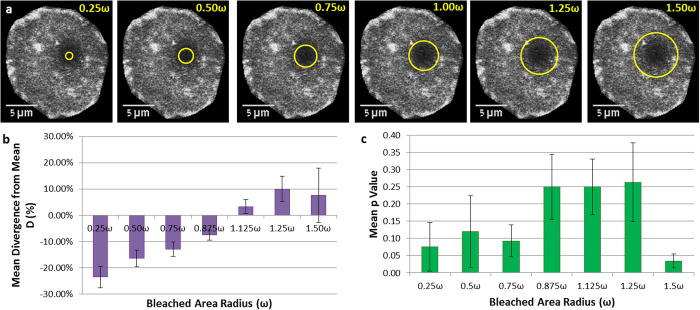
Effects of Selected Monitored Area on D (**a**) Images showing the different radii of selection circles, from 0.25 ω to 1.5 ω overlaid on the bleached area. (**b**) Mean Divergence of D as a function of selected monitored area radius (0.25 ω–1.5 ω). The calculated diffusion coefficient deviates by 10% to 20% from the value for “correct” choice of the area (radius = ω) for a range of ~0.25 ω < r < ~1.50 ω. (**c**) However, the deviations for the range ~0.85 ω < r < ~1.25 ω are statistically insignificant (p > 0.1), constituting a “safe” space for user errors. Error bars represent SEM.

In our implementation of the algorithm, the only user-dependent input is the selection of the bleached area, the area that is used to extract the recovery curve from the time-lapse stack of images. To investigate the sensitivity of our method to this somewhat arbitrary choice, we ran the simulation on the circular beam bleaching data sets, with various selected apparent bleached areas (concentric with the actual bleached area, but of various radii), as demonstrated in Fig. 5a. We report the results in a normalized fashion, where the radius of the selection circle is given in units of ω, the extracted Gaussian beam radius. The resulting diffusion coefficient is reported as deviation from the “correct” D, i.e. the one resulting for the choice of a circle of radius ω (*D*_*ω*_). Each value of D is the average of 10 independent runs with the same radius of selection circle.

Results of this analysis on seven separate series are described in Fig. 5b. A clear trend is observed, where the apparent diffusion coefficient depends monotonically on the radius of the chosen area. An apparently smaller than correct area should recover faster (due to smaller diffusion distances required) than the truly bleached area is actually recovering. Therefore the apparent diffusion coefficient for the specified (too small) area is low (slow). Conversely, a larger than correct monitored area should recover slower (due to larger diffusion distances) than the truly bleached area is actually recovering, thus the apparent diffusion coefficient seems faster. Thus it is obvious that choosing the correct monitored area is important, if the correct diffusion coefficient is sought. This fact is obviously true for the classical approaches as well, and is analogous to the correct determination of ω in the Axelrod method, in the absence of which, the reported *D* values are erroneous. Moreover, the classical method is extremely sensitive to the correct determination of ω, both due to experimental difficulties and the square power dependence of the diffusion coefficient on ω (see equation (3) and accompanying text in the supplementary material). To investigate the sensitivity of our method to wrong choice of the monitored area, we calculated the significance (p-value) of the deviation from the correct *D* value, and plotted the results in Fig. 5c. For choices of too small (0.25 ω to 0.75 ω) an area, or too large (1.5 ω) one, the deviation of *D* from the correct value is unacceptably significant (p < 0.1); however for moderate errors in selecting the monitored area (~0.85 ω < r < ~1.25 ω) the resulting *D* values are insignificantly different (p > 0.1) from the correct values. In light of the large commonly encountered cell-to-cell variation of *D* (up to an order of magnitude) for a single experiment, a severe error in correct choice of the monitored area (less ~0.75 ω or more than 1.5 ω) will result in a significant deviation, however even that is a mere 10% off the correct value, showing this approach is relatively insensitive to user errors. The range of 0.875 ω < r < 1.25 ω in user-selected bleached area can thus be considered “safe”. Despite this relatively low sensitivity to wrongly selected bleached area, efforts should be made to correctly select it, and we suggest experimental guidelines for the correct choice in the Supplementary material).

## Discussion

Commercially available modern microscopes allow easy FRAP experiments to be conducted by virtually anyone. The ease of use and accessibility, however, take a toll (in fact, two):

1. If basic assumptions underlying the extraction of diffusion coefficients are violated (such as bleaching too large areas of cells), erroneous values of D will be obtained, as demonstrated in supplementary figure S2. No alert will be given to the uninitiated user.
2. If arbitrary bleaching geometries are used (other than rectangular boxes or circular areas), the extracted D values are meaningless and wrong, because no analytical solutions exist for arbitrary boundary conditions of the diffusion equation.

Still, keeping the initial and boundary conditions constant between experiments may allow comparative studies, based on observation of higher or lower mobility of compared species; this type of studies is widely performed and reported in the literature; however it is not possible to provide an absolute value of the diffusion coefficient (D).

How important is it to measure D in absolute rather than in relative units? It is as important as measuring heights, for example, in absolute units. Though it is possible to compare heights in terms of “taller” and “shorter” and reach important conclusions based on such comparative studies, the importance and added value of extracting numerical, absolute values of height (in whatever unit of length) is obvious. Extracting absolute values of D (as opposed to relative values) similarly is important in a number of cases. One case is the basic calibration of the measurement system, where a sample with known (from other methods) diffusion coefficient is measured with the system under validation. If an absolute value of D cannot be extracted, there is no way of confirming that the system measures correctly the entity it is supposed to measure. In other cases, an absolute D value may directly confirm the identity of the studied sample. For example, D for membrane lipids usually does not deviate by one order of magnitude from the known values. If it does, the sample under investigation may not be a lipid, the probe may interact with membrane proteins, or the choice of an inappropriate size and shape of bleached area may have yielded recovery curves not fit by the usual diffusion equations. Absolute values of D are also useful in connecting FRAP measurements to results for D from single particle or single molecule tracking (SPT) approaches. In contrast to FRAP, which yields an average diffusion coefficient, SPT techniques yield a distribution of diffusion coefficients, built-up from individual diffusion coefficients of single particles. If FRAP cannot yield an absolute value of (mean) D, there is no way to correlate FRAP and SPT experiments.

The method presented here takes advantage of modern tools, fast imaging devices and powerful personal computers, to extend the spectrum of possible applications of FRAP to a wide community of scientists. The free availability of the algorithm should facilitate quantitative FRAP measurements for many users equipped with standard fluorescence microscopy setups. Our approach eliminates the need for tedious characterization of the bleaching geometry and depth, and requires merely a stack of time-lapse images and rough selection of the bleached area, for an arbitrary bleaching geometry. Moreover, slight errors in characterization of bleaching parameters required for the classical approach may introduce large errors in determination of diffusion coefficient, an effect avoided by the simulation approach reported here.

The approach is validated using a well characterized diffusing molecule (DiIC_18_) against well-established analytical procedures, and is shown to yield statistically similar results, both for geometries compatible with the analytical methods, and for arbitrary bleaching geometries. The results are robust in terms of stochastic fluctuations resulting from the simulation approach, and relatively insensitive to the user-selected apparent bleached area.

This algorithm assumes pure diffusion only, however the concept can be extended to more complex modes of diffusion, such as sub- and super-diffusion, binding-reaction diffusion, etc.

## Methods

### Cell Culture

All reagents were purchased from Biological Industries (Kibbutz Beit Haemek, Israel) unless otherwise noted. Normal mouse fibroblasts (ATCC CCL-1.3) were cultured in 250 ml TC flasks (Cellstar, GrenierBio-One, Frickenhausen, Germany) at 37 °C and 5% CO_2_ in Dulbecco’s Modified Eagle Medium (DMEM) with L-glutamine, 10% fetal bovine serum and 1% Pen-strep solution. For mounting, flasks were washed with 10 ml of phosphate buffer saline (PBS) and then incubated in 2 ml trypsin EDTA 0.25% at room temperature until detachment from the flask (about 2 min). 7.5 × 10^5^ cells were plated on homemade glass bottom (#1 cover-slide) 50 mm diameter Petri dish, in 5 ml medium, and incubated overnight.

### Staining

0.934 mg of 1,1_-dioctadecyl-3,3,3′,3′-tetramethylindocarbocyanine perchlorate (DiI), specifically DiIC_18_(3) (Molecular probes, Eugene, Oregon, USA) were dissolved in 1 mL of Ethanol to prepare a 1 mM Stock solution. For staining of cells, a 2.5 μM staining solution was prepared by adding 2.5 μL bulk solution to 1 mL serum-free growth medium. Following overnight incubation, growth medium was removed from Petri dishes and 500 μL of DiI staining solution were added, followed by 1 hour incubation at 0 °C. The dishes were then washed 3 times with 5 ml normal growth medium, allowing for 5 minutes incubation at 37 °C and 5% CO_2_ between washes. Before imaging, growth medium was removed and dishes were gently rinsed 3 times with 5 ml warm (37 °C) Hepes Hank’s balanced salt solution.

### Imaging

Cells were imaged with a Hamamatsu C9100-50 cooled EM-CCD camera on an Axiovert-200 M microscope (Zeiss, Germany) with Plan-Neofluar 63x/NA 1.4 objective. The microscope is fitted with a UltraView ERS FRET-H- Spinning Disc Confocal system (Perkin-Elmer, Waltham, MA, USA) with a FRAP module. DiI was bleached using the 488 nm laser line from an Argon ion laser, with varying bleaching time (depending on bleaching pattern and cell intensity).

## Additional Information

**How to cite this article**: Blumenthal, D. *et al.* Universal Approach to FRAP Analysis of Arbitrary Bleaching Patterns. *Sci. Rep.* **5**, 11655; doi: 10.1038/srep11655 (2015).

## Supplementary Material

Supplementary Information
