# Supplementary material for: Universal Approach to FRAP Analysis of Arbitrary Bleaching Patterns
Source: Sci Rep. 2015 Jun 25;5:11655. doi: 10.1038/srep11655 (PMC4479983; doi:10.1038/srep11655)
Supplement: Supplementary Information [file srep11655-s1.pdf]

`

Universal Approach to FRAP Analysis of Arbitrary Bleaching Patterns

Daniel Blumenthal†, Leo Goldstien†, Michael Edidin‡ and Levi A. Gheber*†.

†The Avram and Stella Goldstein-Goren Department of Biotechnology Engineering, Ben Gurion University of the Negev, Beer-Sheva, ISRAEL. ‡Department of Biology, Johns Hopkins University, Baltimore, MD, USA

Contact information:
Levi A. Gheber: glevi@bgu.ac.il
Daniel Blumenthal: danielbl@post.bgu.ac.il
Michael Edidin: edidin@jhu.edu
Leo Goldstien: duncang@post.bgu.ac.il

Supplementary Material

Simulation Approach to FRAP Computation and Analysis

Our simulation approach allows computation of diffusion coefficients regardless of bleaching geometry used in the FRAP series. The method is based on fitting a computer-simulated recovery to actual recovery data of a FRAP series. The algorithm accepts a multiple-frame TIFF file, representing the experiment as input, and simulates the (pure) diffusion of the fluorescent probes (2D random walk) starting with the first post-bleach frame of the actual data. Once the simulated recovery is finished, the algorithm fits the simulated data to the real one and extracts the diffusion coefficient.

The algorithm iteratively creates a series of simulated images, where each frame corresponds to a single iteration. The intensity values are extracted from the (user indicated) bleached area of the simulated frames, thus determining the general shape of the recovery curve. The "time" axis at this stage is in arbitrary units (iterations). To extract the diffusion coefficient, the simulated recovery curve needs to be fitted to the real recovery curve, by appropriately stretching the “time” axis. The time between frames in the actual data set is obviously known, thus once overlapping optimally the simulated curve with the real one, the duration of one iteration, in real time units, is determined. The diffusion coefficient of the simulated series is then calculated using

$$(1)\quad \boldsymbol{D_s = \frac{l^2}{4t_i}}$$

where D_s is the simulation-extracted diffusion coefficient, l is the step of a molecule in each iteration of the simulation, corresponding to one pixel in the image (the pixel size is calibrated previously, by imaging a known calibration sample), and t_i is the time interval between steps (determined as explained).

Technically, the simulation proceeds until a plateau is reached (equilibration of the fluorescence intensity in the bleached area). The number of data points in the simulated recovery is typically different (larger) than the number of experimental points. In addition, the real experimental data may not have been acquired until equilibration of fluorescence. For this reason, in order to determine t_i, the algorithm scans a range of possible values for the total duration represented by the simulation and calculates a value (χ^2) for the goodness-of-fit between the simulated data and the real FRAP data. Total simulation

duration is selected as the one that that produces the minimal χ^2. An example of thus simulated data overlapped on real data is shown in Supplementary figure S3a. Supplementary figure S3b shows the goodness-of-fit value as a function of total duration represented by the whole simulation. The sharp minimum occurs for 11.7 seconds (this is the total time represented by the blue set in Supplementary figure S3a). The value of t_i is then calculated by dividing the total simulation duration (determined as above) by the number of iterations.

Traditional Analysis of FRAP Data

Circular Bleaching Pattern

Cells were bleached with beam diameters between 2.4 – 6.0 μm and fluorescence recovery was imaged at rates of 5 – 20 frames per second. A recovery curve was extracted by quantifying the intensity of the bleached area along the stack of images and normalized by dividing each value by the intensity of the same area before bleaching. From the recovery plot, a typical recovery time, τ_D, was extracted by fitting the data with a model in the form of [1]:

$$(2)\ \hat{\boldsymbol{I}}_{(t)} = \sum\left[\frac{-\boldsymbol{K}^{\boldsymbol{n}}}{\boldsymbol{n}!}\right]\left[\mathbf{1} + \boldsymbol{n}\left(\mathbf{1} + \frac{\mathbf{2}\boldsymbol{t}}{\boldsymbol{\tau_D}}\right)\right]^{-\mathbf{1}}$$

where $\hat{I}_{(t)}$ is the fluorescence intensity of the bleached area divided by the pre- bleaching intensity, K is the extent of bleaching, τ_D is the typical recovery time and n is a positive integer. Fitting was done with a series of n=6. To calculate the diffusion coefficient, (D_c) we used [1]:

$$(3)\ D_c = \frac{\omega^2}{4\tau_D}$$

where ω, the radius of the Gaussian bleaching profile at $1/e^2$ height, was extracted from the frame following the bleaching pulse (Supplementary figure S4e,f), and $\tau_D$was from fitting equation (2) to the data.

The most important cause for variation in the calculated value of D is the difficulty to extract a reliable value for ω from the Gaussian fit to the bleached spot. The problem in resolving ω is caused by bleached spots that are asymmetric in both geometry and intensity, as well as random noise in the imaging system. To minimize these, we performed angular averaging of the data around the center of the bleached spot, as described in[2], and shown in Supplementary figure S4

Rectangular Bleaching Pattern

Cells were bleached with rectangular patterns with dimensions between 2.5X2.5 μm^2 to 4X4 μm^2. Calculating the diffusion coefficient for rectangular areas was done by fitting the recovery data to a previously reported model in the form of:

$$(4)\ I_{(t)} = I_{(\infty)}\left(1 - \sqrt{\left(w^2(w^2 + 4\pi D_c t)^{-1}\right)}\ \right)$$

where $I_{(t)}$ is the fluorescence intensity as a function of time, $I_{(\infty)}$ is the intensity for $t \to \infty$ and w is the "width" (somewhat obscure definition, especially for high aspect ratios) of the rectangle. To avoid the confusion associated with the definition of w, we avoided bleaching high aspect-ratio rectangles.

`

Calculating Error Bars

Circular bleaching pattern

According to equation (3), the error in the diffusion coefficient (D_c) stems from two sources. First, error in the estimation of the bleach spot radius ω – reported by the fitting algorithm that fits the bleached spot intensity profile to a Gaussian and second, the error in the estimation of τ_D extracted from fitting FRAP data to equation (2).

Rectangular Bleaching Pattern

D_c was extracted directly from fitting the experimental data with the empirical equation (4). The error is reported by the fitting algorithm.

Simulation

The simulation diffusion coefficient (D_s) was calculated using equation (1). Similarly to the circular bleaching pattern, the error here stems from two sources. First, error in pixel size (l) (provided as input by the user), which was estimated as 10% of the value. Second, error in the estimation of τ_i resulting from fitting different iteration times for the simulation and choosing the one which best fits the real FRAP data. The error range was estimated as the time difference between the values of τ_i adjacent (on the left and right) to the best (chosen) one.

Experimental guidelines for choosing the correct bleached area

First, it is important to acquire high contrast data expressed in a high signal to noise ratio. Particularly important is the background, please make sure that your subject has more fluorescent intensity than the background. Next, bleaching should be carried out to an extent that allowing differentiation between bleached and unbleached areas. If, after taking all these into consideration, there is still doubt on the exact bleached area, it is possible to use an image analysis software (such as ImageJ) to create an intensity profile along the axis of the bleached area, using it to find the exact edge location.

`

REFERENCES:

1. Axelrod, D., Koppel, D.E., Schlessinger, J., Elson, E.L. & Webb, W.W. Mobility Measurements by Analysis of Fluorescence Photobleaching Recovery Kinetics. *Biophysical Journal* 16, A217-A217 (1976).
2. Jonsson, P., Jonsson, M.P., Tegenfeldt, J.O. & Hook, F. A Method Improving the Accuracy of Fluorescence Recovery after Photobleaching Analysis. *Biophysical Journal* 95, 5334-5348 (2008).

SUPPLEMENTARY FIGURE LEGENDS

Supplementary figure S1 – Lack of correlation between bleached area size and diffusion coefficient
Diffusion coefficient extracted from simulation is not correlated (r=0.283, p=0.17) with bleached area dimensions. The different groups, distinguished by their bleaching geometry are marked with different colors. Circular (Gaussian) spots in purple, Box geometry in green and arbitrary shapes in orange.

Supplementary figure S2 – Bleaching an exaggeratedly large diameter circular area
(a,b) Images of a cell prior to, and immediately after bleaching an exaggeratedly large diameter circular laser beam, respectively. (c) Diffusion coefficients resulting from attempting to use the classical, closed-form solution for increasingly large circular areas. When the bleached area is greater than ~25%, the calculation results in errors.

Supplementary figure S3 - Fitting Simulated Data to FRAP Series
(a) Simulated data (blue) and FRAP series (red), for the optimal $\boldsymbol{t_i}$. (b) the $\chi2$ plot showing a minimum for a total duration of simulated recovery of 11.7 s. $\boldsymbol{t_i}$ is this number divided by the number of iterations

Supplementary figure S4 - Effects of Circular Averaging on $\boldsymbol{\omega}$
(a,b) Images of a cell prior to, and immediately after bleaching with a Gaussian laser beam, respectively. (c) Zoom-in of the bleached area in b, highlighted in the yellow square (d) Angular averaging of the bleached area (c). (e,f) Intensity profile along the red dashed lines in images (c) and (d) respectively, with the calculated ω value for each Gaussian fitting (solid lines).

SUPPLEMETARY FIGURES

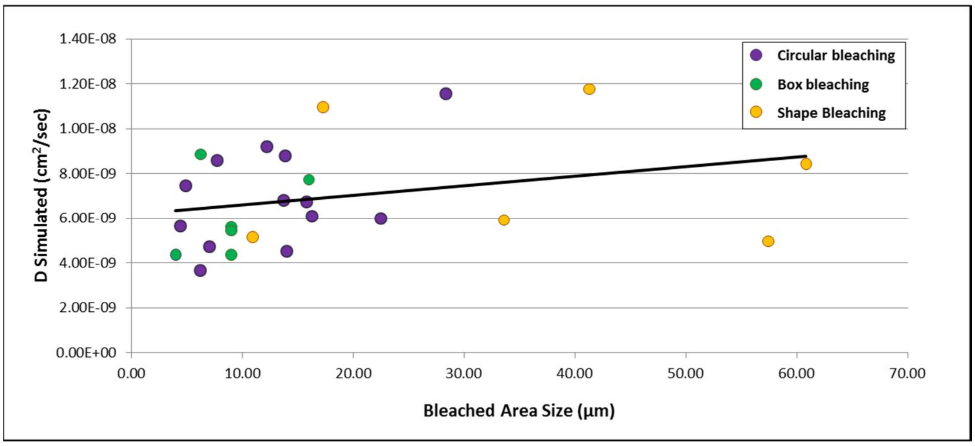

<u>Supplementary figure S1 – Lack of correlation between bleached area size and diffusion coefficient</u>
Diffusion coefficient extracted from simulation is not correlated (r=0.283, p=0.17) with bleached area dimensions. The different groups, distinguished by their bleaching geometry are marked with different colors. Circular (Gaussian) spots in purple, Box geometry in green and arbitrary shapes in orange.

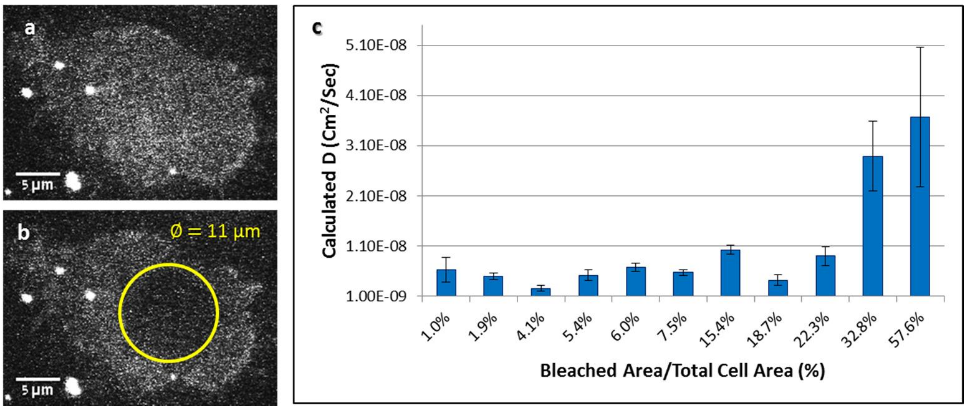

<u>Supplementary figure S2 – Bleaching an exaggeratedly large diameter circular area</u>
(a,b) Images of a cell prior to, and immediately after bleaching an exaggeratedly large diameter circular laser beam, respectively. (c) Diffusion coefficients resulting from attempting to use the classical, closed-form solution for increasingly large circular areas. When the bleached area is greater than ~25%, the calculation results in significant errors.

`

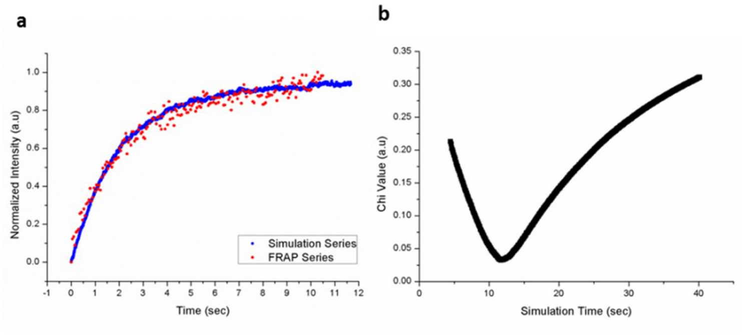

Supplementary figure S3 - Fitting Simulated Data to FRAP Series
(a) Simulated data (blue) and FRAP series (red), for the optimal t_i. (b) the χ2 plot showing a minimum for a total duration of simulated recovery of 11.7 s. t_i is this number divided by the number of iterations

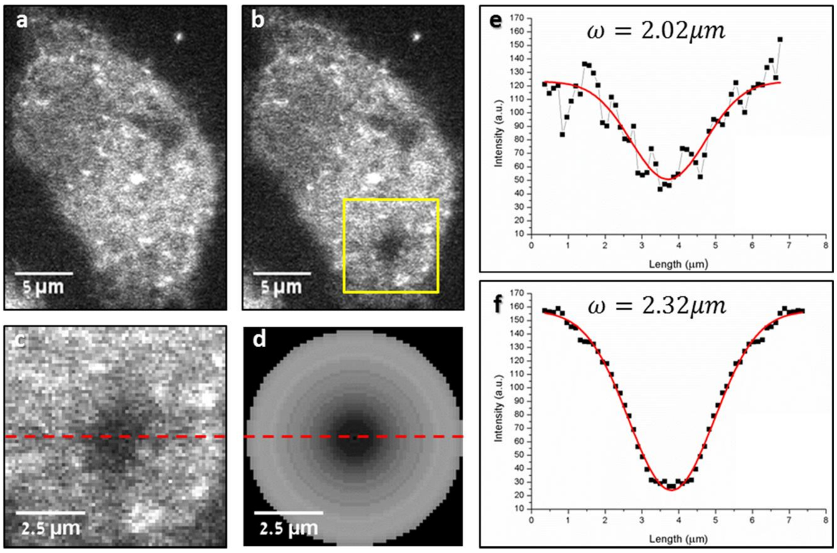

Supplementary figure S4 - Effects of Circular Averaging on ω
(a,b) Images of a cell prior to, and immediately after bleaching with a Gaussian laser beam, respectively. (c) Zoom-in of the bleached area in b, highlighted in the yellow square (d) Angular averaging of the bleached area (c). (e,f) Intensity profile along the red dashed lines in images (c) and (d) respectively, with the calculated ω value for each Gaussian fitting (solid lines).
